# Stimulating existential communication – first steps towards enhancing health professionals' reflective skills through blended learning

**DOI:** 10.1016/j.pecinn.2023.100121

**Published:** 2023-01-07

**Authors:** Connie Timmermann, Christina Prinds, Elisabeth Assing Hvidt, Niels Christian Hvidt, Marianne Engelbrecht Lau, Jette Ammentorp

**Affiliations:** a Centre for Research in Patient Communication, Odense University Hospital, Kløvervænget 12B, 5000 Odense C., Denmark; b Department of Clinical Research, University of Southern Denmark, J.B. Winsløwparken A19, 5000 Odense C., Denmark; c Hospital Sønderjylland, Kresten Philipsens Vej 15, 6200, Aabenraa; d Research Unit for General Practice and Department of Public Health, University of Southern Denmark, J.B. Winsløwparken 19A, 5000 Odense C., Denmark; e Capital Region of Denmark, Denmark

**Keywords:** Reflective practice, Existential communication, Paticipatory action research, Communication training, Reflective skill, Blended learning

## Abstract

**Introduction:**

Talking about existential issues with patients is often experienced as challenging for healthcare professionals. This paper describes our first steps towards developing existential communication training with particular attention to reflective learning methods. Blended learning was chosen to support reflection and an easier transition to classroom conversations, and through Participatory Action Research (PAR), patients were involved in developing the curriculum.

**Method:**

To develop the most valuable and relevant communication training, patients, relatives, healthcare professionals and researchers were involved in a PAR process including 1) three theatre workshops and 2) collaborative meetings to develop the blended learning curriculum and reflection videos. The evaluation of the communication training was based on semi-structured interviews with the healthcare professionals participating in the blended learning communication training.

**Discussion and innovation:**

The results indicate that a blended learning format involving a high degree of reflection is valuable for developing skills related to existential communication. Engaging patients in the process may be essential to develop a training curriculum for healthcare professionals that accommodates the patient's needs.

**Conclusion:**

Future communication training on existential communication may benefit from adopting a blended learning format, including reflective learning methods and the involvement of patients in curriculum development.

## Highlights

• Involving the patients in the development of health communication curriculums is an important way to accommodates the patient's needs.
• Exercises involving a high degree of peer reflection are valuable for developing reflective skills related to existential communication.
• Reflective skills are essential to stimulate the health professionals' consciousness about their pre-understanding and emotions.
• In developing the most valuable communication training involving patients, relatives and healthcare professionals, is essential.

## 1 Introduction

Talking with patients about their existential concerns and resources, such as fear of the future or loss of identity and sources of meaning, is challenging for healthcare professionals [[1], [2], [3]]. Some of the challenges include feelings of uncertainty and vulnerability [[4], [5], [6]]. Based on “whole-person care” and “relational-centred care”, where a human-to-human relationship is emphasized, a common vulnerability is inevitable because all parties bring emotions, common existential conditions and experiences to the encounter [[7], [8], [9]]. Recent literature suggests incorporating reflection into learning when dealing with communication training about existential issues and vulnerable encounters [6,10]. Likewise, studies show that communication skills training combining knowledge building and self-reflection contributes to increased confidence in addressing the patient's existential issues as a health professional [11,12]. Blended learning was chosen to support reflection and the transition to classroom conversations [13]. In addition, the literature emphasises the needs and benefits of involving patients in improving communication between healthcare professionals and patients [3,14]. However, there is still much to learn about how patients can engage more actively in developing content for clinician communication training.

This paper describes our first steps towards developing existential communication training through blended learning. We discuss developing our reflective learning method, which builds healthcare professionals' skills and confidence in discussing existential matters. In addition, we demonstrate one way of involving patients in the development of a communication training curriculum through participatory methods.

## 2 Method

### 2.1 Setting

This study was carried out at a Danish university hospital in 2018 as a follow-up on the large-scale communications program ‘Clear Cut Communication with Patients’ implemented at the hospital between 2011 and 2017. This communication program is based on the Calgary Cambridge Guide and the “train-the-trainer” concept [15].

### 2.2 Design

Participatory Action Research (PAR) was used as a scientific research approach to create active interaction through the interpretation and negotiation of meaning and dialogues between researchers, patients, relatives and clinical practitioners [16]. Inspired by PAR, we used “improvised participatory theatre” to include those most involved in a clinical encounter as the basis for developing the new training course [17,18].

### 2.3 Participatory improvised theatre workshops

Three theatre workshops were conducted to explore communicative encounters between healthcare professionals, patients, and relatives involving barriers, existential issues, and meaning-making resources. The theatre method of improvised participatory theatre has been shown to create valuable cycles of reflections and knowledge about human interactions and the existential dimension in healthcare communication while at the same time involving the patient's experiences [3,18].

Each theatre workshop was initiated based on a clinical case involving existential concerns. The case was transformed into a theatre scenario in collaboration with a dramaturgist, and professional actors then performed this scene as an opening scene. The opening scene formed the basis for further exploration, discussions, and new improvised scenarios [3,18].

The invited participants were patients, relatives, healthcare professionals from clinical practice, trainers from the ‘Clear Cut Communication programme [15] and researchers.

As part of the PAR process, collaborative meetings were held with patients, relatives, clinicians, theatre workshop actors, blended-learning specialists and other researchers working with existential themes in healthcare communication. The purpose was to bring together knowledge and reflections from the theatre workshops as the basis for creating a blended learning curriculum and reflection videos.

### 2.4 The blended-learning course on existential communication

The curriculum of the new training course was blended learning combining in-class training and a digital approach. Before the in-class training, participants were asked to complete the digital learning section, where they were prompted to reflect on our developed reflection videos guided by a reflection questionnaire. The questions allowed for self-reflection, dialogue, and reflection between course participants. The participants were allocated approximately 30 min to complete the digital section.

The in-class training of the blended learning course consisted of three sessions, each lasting four hours.

The in-class curriculum consisted of 1) theoretical input related to existential aspects of illness, 2) practical exercises in smaller groups, and 3) reflection exercises based on, among others, the reflection videos and the “ Sources of Meaning Card-Method” (SoMeCaM – see somecam.org). The SoMeCaM is a method of assessing and exploring sources of personal meaning by working with a peer [19].

### 2.5 Test of the blended-learning course on existential communication

Two course pilot tests were conducted based on semi-structured qualitative interviews with the participants [20].

#### 2.5.1 Participants

Two courses based on the recommended train-the-trainer concept with fourteen participants were held, allowing healthcare professionals to train other healthcare professionals [21]. The first course consisted of seven participants, and they were chosen because they were already trainers in Clear Cut Communication with Patients [15]. The trainers were medical doctors, nurses or physiotherapists from different clinical departments. The newly educated trainers were responsible for teaching the second course consisting of seven clinicians from an orthopaedic surgery department. Again, the clinicians were medical doctors, nurses or physiotherapists who volunteered to try the existential communication course.

#### 2.5.2 Interviews

For pragmatic reasons, telephone interviews were chosen to evaluate the courses, each lasting between 25 and 40 min. All fourteen participants agreed to be interviewed. The first author performed the interviews with the main themes: How did you experience the course in general? How did you experience working with the reflection videos? How did you experience working with the “Sources of Meaning Card Method”?

Brinkmann inspired the analysis on understanding a situation, in this case, the content of interviews, by sense-making [22]. The analysis consisted of re-reading the transcribed interviews to get an overview of the content and understand the text's entirety. The first author conducted the analysis in collaboration with the other authors.

## 3 Results

Overall, the evaluation of the training program was positive. However, it was stressed by the participants that a safe learning environment is crucial when addressing own vulnerabilities and sources of meaning. Patient involvement in the improvised theatre workshops directly influenced the development of the content in the curriculum and the reflection videos. Furthermore, the key elements of the training program facilitating the reflective competencies of the healthcare professionals were the digital reflection videos and the reflection cards (SoMeCaM). The experiences from the first two blended learning courses on existential communication highlighted how the combined methods of individual and peer reflection were valuable for developing the participant's reflective skills on existential issues. The following citations from healthcare professionals attending the existential communication course illustrate this:

“What is it about the patients that ‘triggers’ me? Something is said in one of the videos, but the person's body language says something else. So we spent much time talking about that.”

“It's exciting to talk about why you experience communication differently (a scene from the reflection videos). What was it I saw - and what didn't I see? ”.

Likewise, the following statements emerged during the interviews regarding the use of the SoMeCaM:

“They are excellent. I was amazed at my sources of meaning, and the exercise set many thoughts in motion. You become wiser about yourself. And I think that makes you better at understanding and accommodating other people”.

“Quite amazing - a pleasure. Working with the cards gives rise to good deliberations. It strengthens my ability to accommodate other people and clarifies that there are many ways to interpret - we had several similar cards with the same values, but we interpreted them differently. It gives me a more versatile understanding of the patients, more nuanced. The differences we have - yes, the patients have them too”.

## 4 Discussion and conclusion

### 4.1 Discussion

The digital reflection videos and the SoMeCaM allowed the course participants to reflect upon different ways of interpreting patients' verbal and nonverbal communication and common existential conditions related to sources of meaning. Existing literature shows that existential conversations require that healthcare professionals can use their relational and communicative skills to “see” and “meet” the patients [23]. Such skills include empathy, picking up verbal and nonverbal allusions, and responding appropriately [24]. However, engaging as a health care professional in conversations that affect the patient's existential vulnerability may also have implications for the professional's experiences of their own vulnerabilities. In other words, a human-to-human relationships affected by a mutual existential vulnerability may influence the communicative encounter [[7], [8], [9]]. The reflective learning approaches developed in our study do not offer a guidebook for talking about existential issues and engaging in vulnerable encounters. Instead, they ask for reflections and personal courage to develop communicative and relational skills. In this way, reflective skills contribute to the healthcare professionals' consciousness about their pre-understanding, emotions, and sources of meaning [6].

A strength of the blended learning approach is that the videos with associated questions provoke reflection and are watched before entering the in-class sessions [25]. This eases the participant's transition to classroom conversations about existential concerns and resources [13]. Moreover, the digital section can be accessed alone or with a colleague.

### 4.2 Innovation

Involving the patients in developing blended learning communication curriculums for healthcare professionals is essential to ensure the completed education accommodates the patient's needs. A way to ensure active engagement through common interpretation and negotiation of meaning is improvised participatory theatre. Through theatre workshops, common reflections and knowledge can become the foundation for developing blended-learning communication curriculums, including reflective learning methods.

### 4.3 Conclusion

The lesson learned from involving patients, relatives and healthcare professionals through participatory methods will inform our future development of blended learning courses. Future communication training to build confidence and skills regarding existential conversations may benefit from adopting reflective learning methods in a blended learning format and involving patients in the process through participatory methods such as improvised participatory theatre.

## Declaration of Competing Interest

The authors declare no conflict of interest.
